# LncRNA TRHDE-AS1 inhibit the scar fibroblasts proliferation via miR-181a-5p/PTEN axis

**DOI:** 10.1007/s10735-021-09968-y

**Published:** 2021-03-06

**Authors:** Yanping Wei, Tingting Wang, Ningning Zhang, Yunyun Ma, Siji Shi, Ruxing Zhang, Xianzhao Zheng, Lindong Zhao

**Affiliations:** 1 Department of Dermatology, People’s Hospital of Jiaozuo City, Jiaozuo, 454002 China; 2 Xinxiang Medical University, Xinxiang, 453003 China; 3 Henan Medical College, Zhengzhou, 451191 China; 4 Department of Neurology, The Fifth People’s Hospital of Jiaozuo, Jiaozuo, 454000 China; 5 Department of Neurology, People’s Hospital of Jiaozuo City, Jiaozuo, 454002 China

**Keywords:** Hypertrophic scar, LncRNA TRHDE-AS1, Fibroblasts, miR-181-5p, PTEN

## Abstract

Hypertrophic scar (HS), a fibroproliferative disorder caused by abnormal wound healing after skin injury, which is characterized by excessive deposition of extracellular matrix and invasive growth of fibroblasts. Recent studies have shown that some non-coding RNA implicated the formation of HS, but the mechanism remains unclear. In this study, we found that lncRNA TRHDE-AS1 was downregulated in HS tissues and HSFs, and the level of lncRNA TRHDE-AS1 negatively correlated with the level of miR-181a-5p in HS tissue and HSFs. Overexpressed lncRNA TRHDE-AS1 significantly suppressed miR-181a-5p level, while promoted HSFs apoptosis and inhibited HSFs proliferation. Further study shown that PTEN was a direct target of miR-181a-5p, and lncRNA TRHDE-AS1 served as a molecular sponge for miR-181a-5p to regulate the expression of PTEN. Overexpression of PTEN could eliminate lncRNA TRHDE-AS1-mediated proliferation suppression of HSFs. In conclusion, our study suggested that lncRNA TRHDE-AS1/miR-181a-5p/PTEN axis plays an important role in promoting hypertrophic scar formation, which may be effectively used as a therapeutic target for hypertrophic scar treatment.

## Introduction

Hypertrophic scar (HS) is a fibroproliferative disorder caused by dermal trauma or severe burn injury, and is characterized by excessive deposition of extracellular matrix and invasive growth of fibroblasts (Aarabi et al. 2007; Zhu et al. 2013). Although it is a non-malignant disease, hypertrophic scar fibroblasts (HSFs) exhibit malignant figures such as excessive deposition of collagen, excessive proliferation, apoptosis resistance, and atypical differentiation (Lim et al. 2006). However, the specific molecular mechanism is not fully understood (Atiyeh 2007). HS can cause physical, psychological, and social discomfort that seriously impairs the quality of life (Zielins et al. 2014). Present treatments for HS including surgical excision, laser removal, silicone gel application, pressure dressing, and steroid injections lack efficacy (Hsu et al. 2017; Willows et al. 2017; Amini-Nik et al. 2018). Therefore, it is necessary to elucidate the mechanism of HS formation and identify novel targets for the treatment of HS.

Long non-coding RNAs (lncRNAs) are non-coding RNAs (ncRNAs) that are more than 200 nucleotides long (Kung et al. 2013). They can act as competing endogenous RNAs to modulate gene expression, inhibit or activate gene expression at transcriptional and post-transcriptional levels, and have been implicated in different biological processes, including cellular proliferation, differentiation, apoptosis, migration, and invasion (Lee 2012; Dong et al. 2018). A recent study has reported that lncRNA TRHDE-AS1 is downregulated in fibroblasts derived from hypertrophic scars (Tu et al. 2018). However, its function in HS needs to be further explored.

MicroRNAs (miRNAs) are single-stranded short ncRNAs with an average length of 22 nucleotides, which perform important cellular functions via regulating the target gene by post-translational repression or degradation of mRNAs (Höbel and Aigner 2013). The miRNAs suppress target gene expression through binding to the 3′-untranslated regions (3′-UTR) of target mRNAs, and consequently influence diverse physiological and pathological processes (Wang et al. 2015; Chen et al. 2017a; Herter and Xu 2017; Wang et al. 2018).

In the present study, we assessed the possible role of the lncRNA TRHDE-AS1 in HS formation and its underlying pathological mechanisms, which provided a novel way of thinking, as well as a theoretical basis for the treatment of HS.

## Material and methods

### Tissue samples

30 pairs of HS tissues and paired adjacent normal skin tissues were obtained from patients who were diagnosed with hypertrophic scars by routine pathological examinations at the Dermatologic Surgery of Jiaozuo People’s Hospital (Jiaozuo, China), from May 2015 to May 2019. All patients were informed of the purpose and procedure of the study, and all patients signed an informed consent. All protocols were approved by the Ethics Committee of the Jiaozuo People’s Hospital. Collected samples were divided into three parts. One was used for the histopathological study after being fixed in 4% paraformaldehyde solution. One was prepared for total RNA and total protein lysates after soaking in liquid nitrogen, and the remaining samples were used for fibroblast isolation and cultures.

### Cell culture

Cultures of primary HSFs and paired normal skin fibroblasts were established as described previously (Liu et al. 2016a). All cells were cultured in Dulbecco’s Modified Eagle’s Medium (Gibco, Gaithersburg, MD, USA) supplemented with 10% fetal bovine serum (Gibco) containing 1% penicillin–streptomycin, and were incubated at 37 °C in a 5% CO_2_ atmosphere. Fibroblasts from the 3rd to 5th passages were used in all experiments unless otherwise indicated.

### Cell transfection

Control pcDNA3.1 plasmid (NC siRNA), expression pcDNA3.1-TRHDE-AS1 plasmids, siRNA (TRHDE-AS1 siRNA, PTEN siRNA), and all synthetic miRNAs, including miR-181a-5p mimic, miR-181a-5p inhibitor, and the respective controls, were purchased from Shanghai GenePharma (Shanghai, China). Lipofectamine 2000 (Invitrogen, Carlsbad, CA, USA) was used for cell transfection according to the manufacturer’s instructions.

### Quantitative reverse transcription-PCR (qRT-PCR)

Total RNA was extracted from cells using TRIzol reagent (Invitrogen) following the manufacturer’s instructions, and the first-strand cDNA was synthesized using a MMLV Reverse Transcriptase Kit (Invitrogen). qRT-PCR was performed using a SYBR Green qPCR Master Mix (TaKaRa, Shiga, Japan). The primer sequences were as follows: lncRNA TRHDE-AS1 forward primer, 5′-CGCTTGTGTACGGCGATGTG-3′, reverse primer, 5′-CTGCTGCGAGCACATTCCAC-3′. The expression of miR-181a-5p was determined using a miRNA qRT-PCR Detection Kit (Tiangen, Beijing, China) according to the manufacturer’s protocol. The primers for miR-181a-5p were as follows: forward primer, 5′-ACACTCCAGCTGGGAACATTCAACGCTGTCGG-3′, reverse primer, 5′-TGGTGTCGTGGAGTCG-3’.

### Cell proliferation assay

Cell proliferation was examined using a CCK-8 assay (Beyotime, Shanghai, China) following the manufacturer’s instructions. A total of 5000 cells were seeded into 96-well plates in triplicate and transfected as indicated for 24, 48, and 72 h, and 10 μL of CCK-8 solution was added to each well. After 4 h of incubation at 37 °C, the absorbance was measured at 450 nm using a microplate reader (Bio-Rad, Hercules, CA, USA).

### Cell apoptosis analysis

After transfection for 24 h, HSFs were digested with 0.25% trypsin, washed twice with cold phosphate-buffered saline, resuspended, and then incubated with annexin V-fluorescein isothiocyanate/propidium iodide in the dark at room temperature for 15 min. Cell apoptosis was performed using flow cytometry. Data were analyzed by the BD LSRFortessa Cell Analyzer (BD Biosciences, San Jose, CA, USA) and FlowJo version 10.0.7 software (FlowJo LLC, Ashland, OR, USA), according to the manufacturer’s instruction. All experiments were repeated three times.

### Western bolt analysis

Total protein from HSFs cells was extracted using Radio Immunoprecipitation Assay (RIPA) lysis buffer. Twenty micrograms of total protein were separated by 10% SDS-PAGE gel and transferred to a nitrocellulose membrane (Invitrogen). The membranes were blocked in 5% nonfat milk at room temperature for 1 h, followed by incubating with specific primary antibodies against PTEN (1:1,000, ab79156; Abcam, Cambridge, MA, USA), and glyceraldehyde 3-phosphate dehydrogenase (GAPDH; 1:2000, sc-47724; Santa Cruz Biotechnology, Santa Cruz, CA, USA) overnight at 4 °C. The membranes were then incubated with horseradish peroxidase-conjugated secondary antibody (1:6000, G-21040; Invitrogen). The protein band were detected using an ECL Western Blotting kit (Thermo Fisher Scientific, Waltham, MA, USA). GAPDH was used as the loading control.

### Luciferase reporter assay

The luciferase reporter assay was performed as previously described (Chen et al. 2017b). The fragment from wild-type lncRNA TRHDE-AS1 containing the predicted miR-181a-5p binding site, mutant lncRNA TRHDE-AS1, the fragment from wild-type PTEN 3′-UTR containing the potential miR-181a-5p binding site, and mutant PTEN 3′-UTR were synthesized by GenePharma and subcloned into the pmirGLO luciferase vector (Promega, Milwaukee, WI, USA). HSFs cells were co-transfected with the above luciferase reporter vectors containing lncRNA TRHDE-AS1 (WT or mutant) or PTEN (WT or mutant) together with the miR-181a-5p mimic, miR-181a-5p inhibitor, or their negative controls (miRNA NC and NC inhibitor) using Lipofectamine 2000 (Invitrogen). After transfection for 48 h, the relative luciferase activity was analyzed by the Dual-Luciferase Reporter Assay system (Promega) according to manufacturer’s instructions. All assays were performed in triplicate.

### Statistical analysis

All data are presented as the mean ± standard deviation (SD). Statistical analysis was performed using Prism 8.0 (GraphPad, San Diego, CA, USA). The Student’s *t*-test or one-way analysis of variance was used to analyze significant differences when only two or more groups were compared. A value of P < 0.05 was considered to be statistically significant.

## Results

### LncRNA TRHDE-AS1 is downregulated in hypertrophic scar tissues

**Fig. 1:**
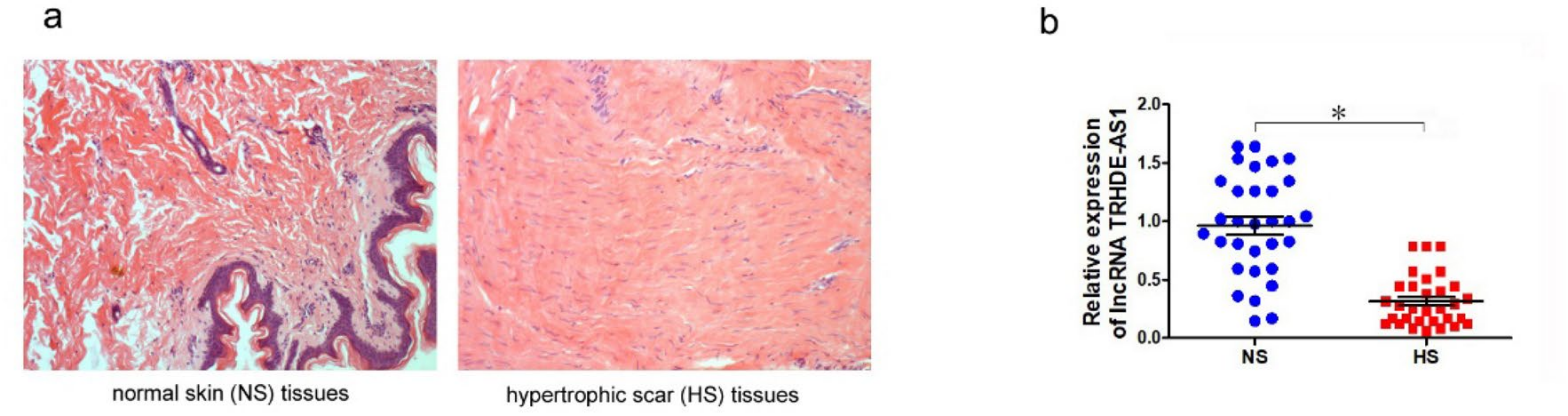
Expression of lncRNA TRHDE-AS1 in hypertrophic scar tissues. **a** Hematoxylin and eosin staining in adjacent normal tissues (NS) and hypertrophic scar tissues (HS). **b** The qRT-PCR analysis of lncRNA TRHDE-AS1expressions in 30 pairs of NS and HS tissues (*P < 0.05)

The hypertrophic scar tissues and adjacent normal tissues were confirmed by hematoxylin and eosin staining after being collected from patients (Fig. 1a). We then examined the expression of lncRNA TRHDE-AS1 in 30 pairs of hypertrophic scar tissues and adjacent normal tissues using qRT-PCR. The results showed that the expression of lncRNA TRHDE-AS1 was significantly decreased in HS tissues when compared to corresponding normal tissues (NS) (Fig. 1b).

### Upregulated lncRNA TRHDE-AS1 suppresses proliferation and promotes apoptosis in hypertrophic scar fibroblasts (HSFs)

**Fig. 2:**
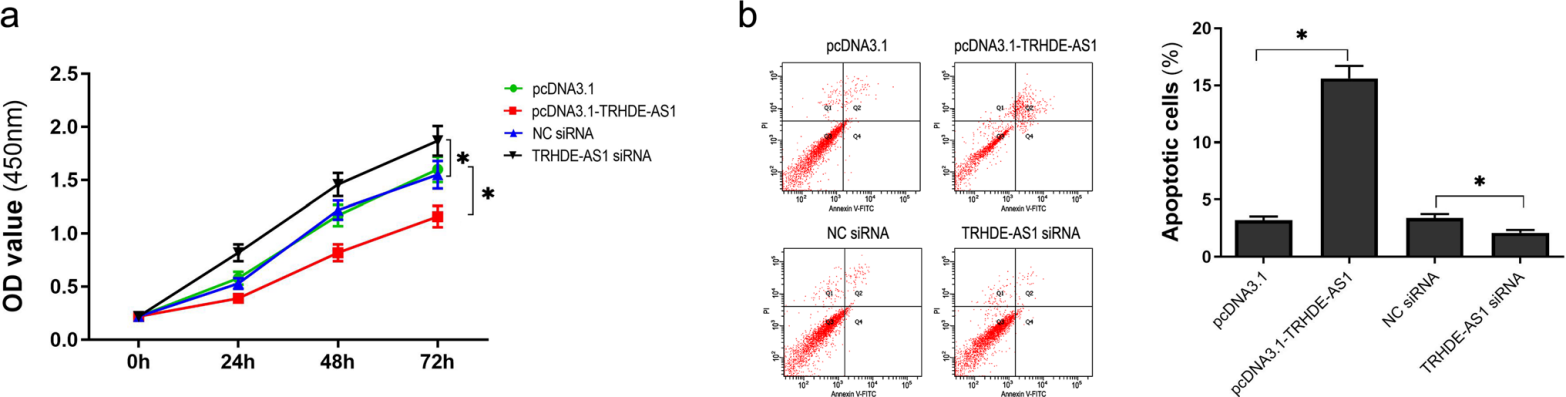
LncRNA TRHDE-AS1 affects the proliferation and apoptosis of HSFs. **a** Cell proliferation ability of HSFs after transfection with pcDNA3.1, pcDNA3.1-TRHDE-AS1, NC siRNA, and TRHDE-AS1 siRNA for 24, 48, and 72 h, respectively, measured by using the CCK-8 assay. **b** Flow cytometry measurement of HSF cell apoptosis and quantification after transfection with pcDNA3.1-TRHDE-AS1 or TRHDE-AS1 siRNA and their respective controls (*P < 0.05)

To assess the effects of lncRNA TRHDE-AS1 on proliferation and apoptosis of HSFs, we measured cell proliferation using the CCK-8 assay and flow cytometry after upregulating or downregulating the expression of lncRNA TRHDE-AS1. Figure 2a shows that overexpression of lncRNA TRHDE-AS1 significantly inhibited cell proliferation; however, it suppressed lncRNA TRHDE-AS1-mediated cell proliferation. Flow cytometry was used to determine the apoptosis of HSFs. The results showed that upregulation of lncRNA TRHDE-AS1 enhanced apoptosis of HSFs. Conversely, downregulation of lncRNA TRHDE-AS1 induced a significant decreased in apoptosis of HSFs (Fig. 2b). These results indicated that overexpression of lncRNA TRHDE-AS1 inhibited cell proliferation and promoted cell apoptosis, suggesting a critical role for lncRNA TRHDE-AS1 in HSF growth.

### LncRNA TRHDE-AS1 sponges miR-181a-5p to inhibit cell proliferation and promote apoptosis in HSFs

**Fig. 3:**
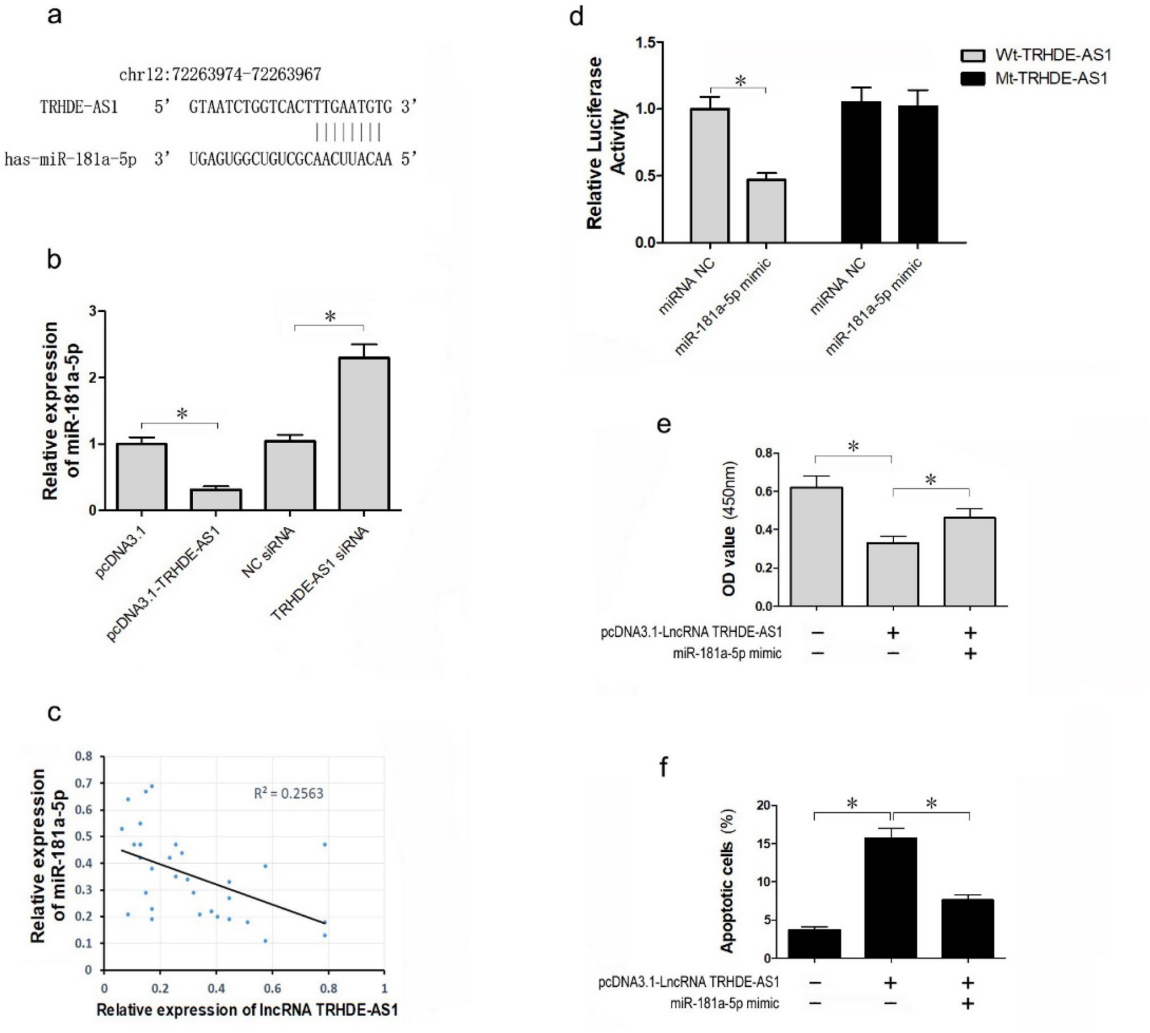
LncRNA TRHDE-AS1 sponges miR-181a-5p to inhibit proliferation and promote apoptosis in HSFs. **a** The predicted binding site of miR-181a-5p to the lncRNA TRHDE-AS1 sequence. **b** The mRNA expression of miR-181a-5p was detected by qRT-PCR in HSFs for overexpression or knockdown of lncRNA TRHDE-AS1. **c** The expression of miR-181a-5p was detected in 30 hypertrophic scar tissues compared to normal control tissues. **d** The luciferase reporter gene assay was performed to confirm the direct binding between lncRNA TRHDE-AS1 and miR-181a-5p. **e** HSFs were transfected into pcDNA3.1-TRHDE-AS1 or control vector, together with (or without) the miR-181a-5p mimic. The cell proliferation was examined using the CCK-8 assay. **f** HSFs were transfected into pcDNA3.1-TRHDE-AS1 or the control vector, together with or without the miR-181a-5p mimic. Cell apoptosis was examined by flow cytometry analysis (*P < 0.05)

Previous studies suggested that lncRNA acts as a sponge for miRNA (Xiao et al. 2015). To investigate the role of lncRNA TRHDE-AS1-mediated proliferation and apoptosis in HSFs, we predicted the miRNA target sites using LncBase (http://carolina.imis.athena-innovation.gr/diana_tools). Figure 3a shows that the bioinformatics analysis revealed that lncRNA TRHDE-AS1contained one conserved target site of miR-181a-5p. To confirm whether lncRNA TRHDE-AS1 regulated miR-181a-5p expression, we investigated the upregulated or downregulated effect of lncRNA TRHDE-AS1 on miR-181a-5p HSFs. qRT-PCR analysis showed that miR-181a-5p expression was inhibited when lncRNA TRHDE-AS1 was overexpressed (Fig. 3b). However, knockdown of lncRNA TRHDE-AS1 upregulated the expression of miR-181a-5p (Fig. 3b). Our qRT-PCR results also showed that the level of lncRNA TRHDE-AS1 was negatively correlated with the level of miR-181a-5p in 30 HS tissues (Fig. 3c).

To confirm whether lncRNA TRHDE-AS1 interacted with miR-181a-5p, a fragment of lncRNA TRHDE-AS1 was inserted downstream of the luciferase gene and a dual-luciferase reporter assay was performed to test the relationship between lncRNA TRHDE-AS1 and miR-181a-5p. The luciferase activity of wild-type lncRNA TRHDE-AS1 was significantly reduced when co-transfected with the miR-181a-5p mimic (Fig. 3d). To further determine whether there was a direct interaction between miR-181-5p and the lncRNA TRHDE-AS1 putative binding site, we mutated the miR-181a-5p binding site, and found that the lncRNA TRHDE-AS1 mutation abrogated the repressive effect of miR-181a-5p (Fig. 3d). These results showed that lncRNA TRHDE-AS1 connected miR-181a-5p directly as a molecular sponge and inhibited miR-181a-5p expression.

To examine if miR-181a-5p was involved in the lncRNA TRHDE-AS1-mediated suppression of cell proliferation and promotion of cell apoptosis, HSFs were transfected with the lncRNA TRHDE-AS1 expression vector alone or with the miR-181a-5p mimic. The overexpression of miR-181a-5p rescued the effect of lncRNA TRHDE-AS1 inhibition on HSF cell proliferation (Fig. 3e). Moreover, the flow cytometry analysis showed that upregulation of miR-181a-5p overcame the effect of lncRNA TRHDE-AS1 apoptosis promotion on HSFs (Fig. 3f). Taken together, these data showed that lncRNA TRHDE-AS1 inhibited HSF proliferation and promoted HSFs apoptosis by inhibiting miR-181a-5p expression, acting as an endogenous sponge to adsorb miR-181a-5p.

### The miR-181a directly targets PTEN in HSFs

**Fig. 4:**
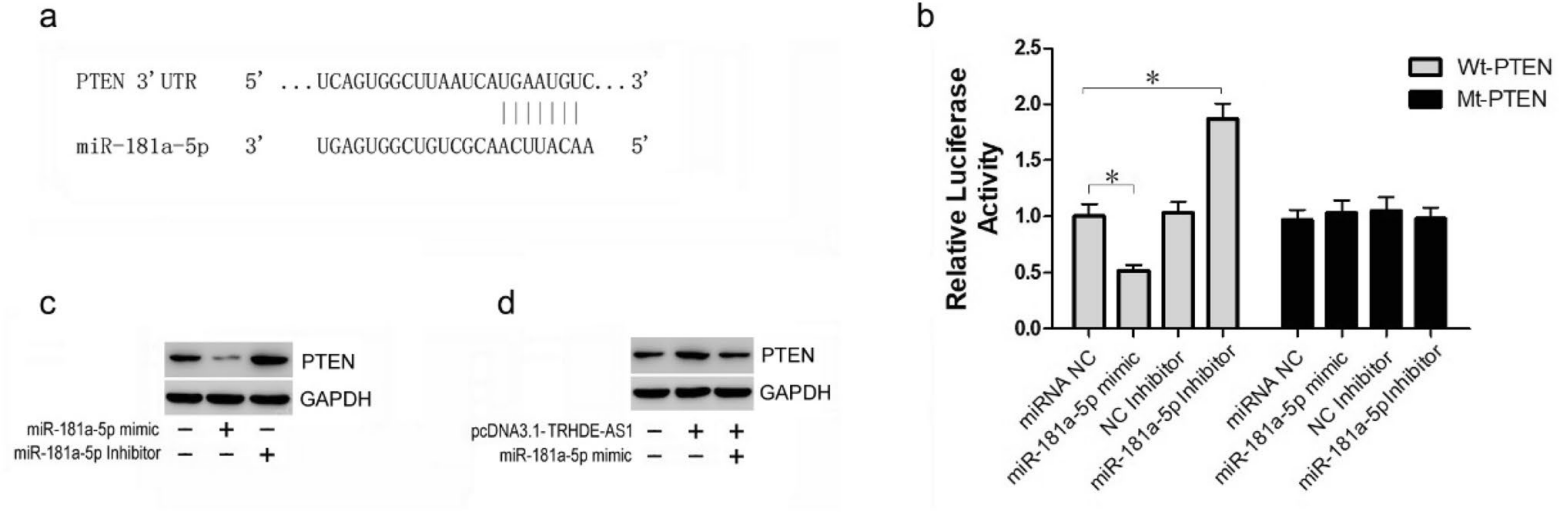
PTEN is the target gene of miR-181a. **a** The predicted miR-181a-5p binding site in the 3′-UTR of PTEN. **b** Hypertrophic scar fibroblasts (HSFs) were transfected with luciferase reporter vector containing wild-type PTEN or mutant PTEN, with or without the miR-181a-5p mimic or miR-181a-5p inhibitor. The luciferase reporter gene assay was used to evaluate the interaction between miR-181a-5p and PTEN. **c** The protein level of PTEN in HSFs transfected with the miR-181a-5p mimic or miR-181a-5p inhibitor. **d** The protein level of PTEN in HSFs transfected with pcDNA3.1-TRHDE-AS1 or the control vector, together with or without the miR-181a-5p mimic (*P < 0.05)

We predicted the target genes of miR-181a-5p by using TargetScan (http://www.targetscan.org). Figure 4a shows that PTEN was predicted to contain the binding sequence of miR-181a-5p. The luciferase reporter assay was then performed. Figure 4b shows that the luciferase activity of wild-type PTEN 3′-UTR was significantly repressed after miR-181a-5p mimic transfection, and significantly increased after miR-181a-5p knockdown. In contrast, the mutant construct completely abolished this effect (Fig. 4b), suggesting that miR-181a-5p directly bound to the PTEN 3′-UTR to inhibit PTEN expression. Western blot analysis showed that overexpression of miR-181a-5p reduced the level of PTEN. However, inhibition of miR-181a-5p increased the protein expression of PTEN in HSFs (Fig. 4c). We then determined whether lncRNA TRHDE-AS1 might regulate the level of PTEN through upregulating miR-181a-5p expression. Western blot analysis showed that increased expression of PTEN caused by lncRNA TRHDE-AS1 overexpression was partially rescued by the miR-181a-5p mimic (Fig. 4d), suggesting that lncRNA TRHDE-AS1 enhanced PTEN expression through negative modulation of miR-181a-5p.

### PTEN mediates the effects of lncRNA TRHDE-AS1 and miR-181a in HSFs

**Fig. 5:**
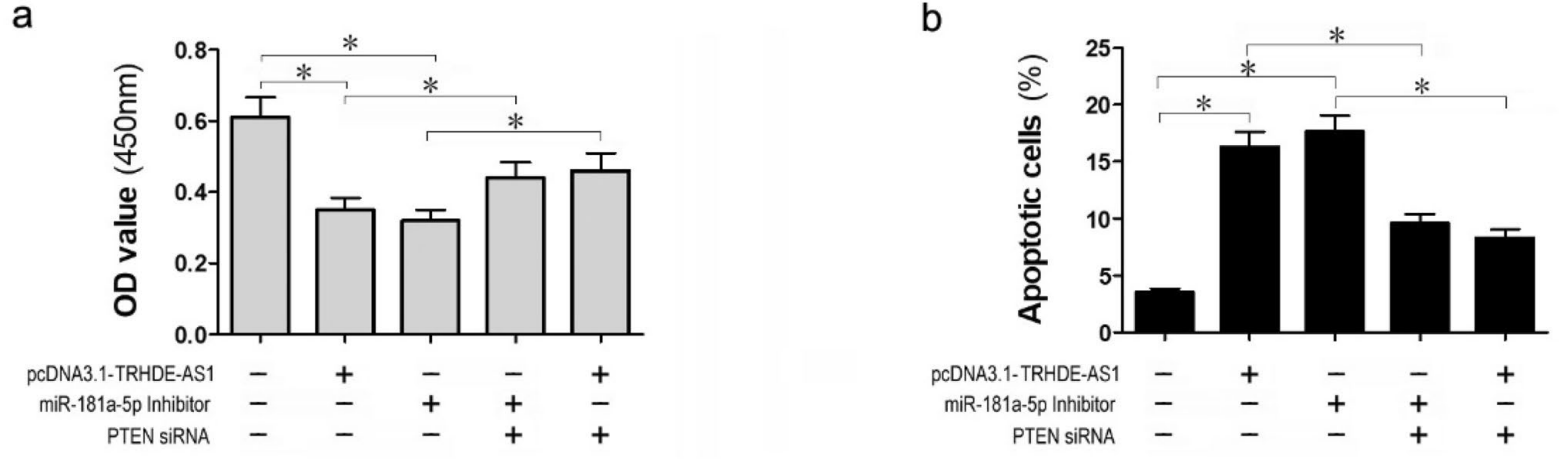
LncRNA TRHDE-AS1 inhibits proliferation and promotes apoptosis of HSFs via regulating the miR-181a-5p /PTEN pathway. After transfection with pcDNA3.1-TRHDE-AS1 or miR-181a-5p, together with or without PTEN siRNA in HSFs, cell proliferation was detected using the CCK-8 assay (**a**) and cell apoptosis was measured by flow cytometry (**b**) (^*^P < 0.05)

We next verified whether the functions of lncRNA TRHDE-AS1 and miR-181a-5p in HSFs were dependent on the expression of PTEN. The CCK-8 assay showed that the suppression of cell proliferation by lncRNA TRHDE-AS1 overexpression or miR-181a-5p knockdown was restored by the suppression of PTEN (Fig. 5a). Similarly, flow cytometry analysis also showed that the apoptosis promotion effect on HSFs induced by lncRNA TRHDE-AS1 overexpression or miR-181a-5p inhibition was suppressed by knockdown of PTEN (Fig. 5b). These results suggested that PTEN mediated the effects of lncRNA TRHDE-AS1 and miR-181a-5p on HSFs, and further confirmed the existence of the lncRNA TRHDE-AS1/miR-181a-5p/PTEN axis in HSFs.

## Discussion

HS is one of the most common skin disorders occurring after thermal injury and trauma, and its formation is a complex biological process involving many cell signaling pathways. It is commonly accepted that fibroblasts are the key participants in HS formation. The aberrant proliferation of fibroblasts and excessive deposition of collagen result in scar formation (Wang et al. 2008; Honardoust et al. 2012). Growing evidence shows the significant differential expression levels of miRNAs and lncRNAs between HS and normal tissues, which further confirm that ncRNAs might play an important role in HS pathogenesis (Yu et al. 2015; Chen et al. 2017a). Tu et al. (2018) used a microarray assay to show that lncRNA TRHDE-AS1 was downregulated in HSFs, which suggested the important role of lncRNA TRHDE-AS1 in HS formation. In the current study, we found significant suppression of lncRNA TRHDE-AS1 in HS tissue and HSFs, compared with normal skin tissue and fibroblasts. In addition, overexpression of lncRNA TRHDE-AS1 promoted HSF proliferation and inhibited apoptosis.

LncRNAs might exert their functions as competing endogenous RNAs (ceRNAs) (Dong et al. 2018). LncRNAs have many binding sites on miRNAs, are termed “miRNA sponges,” and act as a competitive miRNA inhibitor, which blocks miRNAs from binding to target genes (Salmena et al. 2011; Zhang et al. 2019). However, miRNAs bind to the 3′-UTR of target mRNAs, which leads to post-translational repression or degradation of the target mRNA (Ha and Kim 2014). It has been accepted that the usual method of regulating human protein-coding genes involves miRNAs (Friedman et al. 2009). In our current study, we found that the level of miR-181a-5p negatively correlated with the level of lncRNA TRHDE-AS1 in HS tissue and HSFs, and bioinformatics analysis showed that there was a conserved target site of miR-181a-5p in lncRNA TRHDE-AS1. The luciferase report gene assay confirmed that miR-181a-5p directly bound to lncRNA TRHDE-AS1. Overexpression of miR-181a-5p attenuated lncRNA TRHDE-AS1-mediated proliferation suppression or apoptosis promotion of HSFs. These results supported the interaction of lncRNA TRHDE-AS1 and miR-181a-5p, and the participation of these components in hypertrophic scar formation.

Recent studies show that miR-181a-5p is multifunction and is involved in diverse cellular functions, including growth, proliferation, death, invasion, as well as tumor suppression and carcinogenesis (Jianwei et al. 2013; Sun et al. 2015; Liu et al. 2016b; Feng et al. 2018). Rang et al. (2016) found that miR-181a promoted cell proliferation and inhibited apoptosis by suppressing PHLPP2 expression, resulting in AKT pathway activation, and consequently accelerating the formation of pathological scars. In order to further assess the functions of miR-181a-5p in the current study, we predicted that PTEN was the target gene of miR-181a-5p using bioinformatics software analyses.

PTEN is a tumor suppresser and has key functions on the cell cycle, apoptosis, proliferation, and migration (Carnero et al. 2008; Chen et al. 2018). The absence of PTEN is thought to be involved in HS pathogenesis, and its downregulation leads to PTEN/AKT pathway activation (Guo et al. 2012; He et al. 2019). The results of our study, using a dual-luciferase reporter gene assay, confirmed that miR-181a-5p bound to PTEN mRNA directly and downregulated PTEN expression, whereas downregulation of PTEN attenuated lncRNA TRHDE-AS1 overexpression, miR-181-5p suppression-mediated proliferation suppression, or apoptosis promotion of HS. Together, these results suggested that lncRNA TRHDE-AS1 interacted with miR-181a-5p directly and induced HS formation by regulating PTEN expression.

In conclusion, we showed that low expression of lncRNA TRHDE-AS1 promoted proliferation and inhibited apoptosis of HSFs by increasing miR-181a-5p levels and suppressing PTEN expression. Our findings provide a new mechanism for the functions of lncRNA TRHDE-AS1 and its binding of miRNA in HS formation, suggesting that the lncRNA TRHDE-AS1/miR-181a-5p/PTEN pathway may be a novel target for hypertrophic treatment.
